# UAVRM-A*: A Complex Network and 3D Radio Map-Based Algorithm for Optimizing Cellular-Connected UAV Path Planning

**DOI:** 10.3390/s25134052

**Published:** 2025-06-29

**Authors:** Yanming Chai, Yapeng Wang, Xu Yang, Sio-Kei Im, Qibin He

**Affiliations:** 1 Faculty of Applied Sciences, Macao Polytechnic University, Macao SAR 999078, China; yanming.chai@mpu.edu.mo (Y.C.); xuyang@mpu.edu.mo (X.Y.); p2316021@mpu.edu.mo (Q.H.); 2 Macao Polytechnic University, Macao SAR 999078, China; marcusim@mpu.edu.mo

**Keywords:** Unmanned Aerial Vehicle (UAV), path planning, radio map, complex network, A-star

## Abstract

In recent research on path planning for cellular-connected Unmanned Aerial Vehicles (UAVs), leveraging navigation models based on complex networks and applying the A* algorithm has emerged as a promising alternative to more computationally intensive methods, such as deep reinforcement learning (DRL). These approaches offer performance that approaches that of DRL, while addressing key challenges like long training times and poor generalization. However, conventional A* algorithms fail to consider critical UAV flight characteristics and lack effective obstacle avoidance mechanisms. To address these limitations, this paper presents a novel solution for path planning of cellular-connected UAVs, utilizing a 3D radio map for enhanced situational awareness. We proposed an innovative path planning algorithm, UAVRM-A*, which builds upon the complex network navigation model and incorporates key improvements over traditional A*. Our experimental results demonstrate that the UAVRM-A* algorithm not only effectively avoids obstacles but also generates flight paths more consistent with UAV dynamics. Additionally, the proposed approach achieves performance comparable to DRL-based methods while significantly reducing radio outage duration and the computational time required for model training. This research contributes to the development of more efficient, reliable, and practical path planning solutions for UAVs, with potential applications in various fields, including autonomous delivery, surveillance, and emergency response operations.

## 1. Introduction

Cellular-connected UAVs [1], as aerial users integrated into cellular networks, greatly enhance the operational scope of UAVs by enabling low-latency control and information transmission, thus broadening their potential applications in various domains. They have widespread applications in various industries, including aerial photography, inspection, logistics, and communications. In the 3D space, a critical issue determining the effectiveness of cellular-connected UAV is how to ensure real-time connectivity with the ground during mission execution and maintain communication quality and mobile monitoring and positioning while efficiently completing the flight from the starting point to the destination in a cost-effective manner.

Cellular-connected UAVs have high demands for radio environment parameters within a specific area. Fortunately, radio maps can exactly provide relevant precise information, contributing to the precise optimization and design of UAVs. A radio map serves as a spatio-temporal database that provides users within a wireless network service area with information on the wireless network signal environment, network coverage, and historical data. There are two primary types of radio maps. One is the model based on the empirical path loss model approach, which selects different path loss models according to the geographical environment, such as the Okumura–Hata model [2,3] and the COST-231 model [4], to quickly construct the radio map. However, because these path loss models may not align perfectly with real-world conditions, significant deviations can arise between the radio map and the actual environment, severely impacting the map’s accuracy and effectiveness [5]. Although relevant research has proposed methods to correct path loss model parameters based on measured data [6], such solutions still face bottlenecks in terms of prediction accuracy. The other type is based on crowdsourcing data fusion [7], which involves collecting radio parameter data from various geographical locations using a large number of mobile terminals (such as smartphones, UAVs, vehicular terminals, etc.). These data are then integrated into the cloud, and regression techniques are utilized to interpolate and estimate the radio parameters across geospatial areas, thereby constructing a radio map with high accuracy [8]. A common interpolation estimation method is the Kriging-based approach [9], and subsequently, many scholars have incorporated machine learning methods to enhance the accuracy of interpolation estimates [10,11,12,13,14]. Additionally, traditional radio map research has primarily focused on 2D maps. Considering that UAVs operate within a 3D space, research on radio maps for 3D scenarios has gradually emerged. A notable example is the framework for constructing 3D spectrum maps proposed by the Wu team, which involves dividing the 3D space, using UAVs to conduct measurements to construct a series of map images, and employing a “repair” method to fill in the radio parameters for unsampled spaces, thereby constructing a 3D radio spectrum map [15].

**Figure 1:**
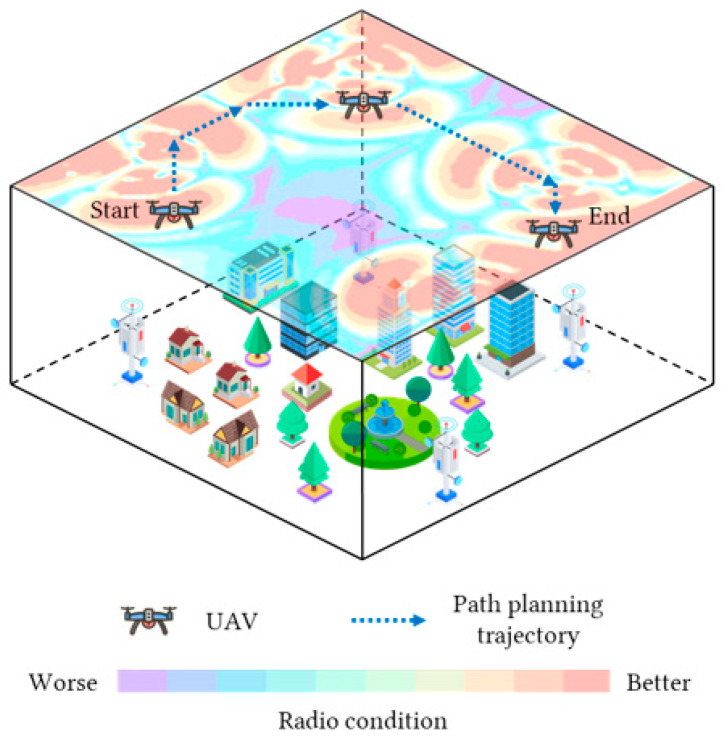
Research scenario of path planning of cellular-connected UAVs in radio map.

Therefore, it is necessary to introduce radio maps into the path planning research of cellular-connected UAV, assisting UAVs in designing paths that can efficiently accomplish tasks while ensuring good network coverage. The research scenario is illustrated in Figure 1. However, despite the abundance of existing studies on UAV path planning, several common limitations persist. Firstly, the majority of these studies still employ overly idealized experimental scenarios, predominantly modeled in 2D planes. Secondly, concerning the algorithms utilized, the most discussed approaches fall into two categories: machine learning and complex network methods. Machine learning (particularly DRL) represents the most mainstream approach. Nevertheless, research in this direction primarily focuses on the path planning performance of the algorithms, overlooking inherent drawbacks in the training process, such as prolonged training times and poor generalization capability of the trained models. Complex network, as a kind of graph theory-based network, can be applied to weighted path planning [16]. Yet, research in this direction inadequately addresses the discussion of navigation models; their structural configurations are overly simplistic, the weight assignments for paths are often arbitrary and unrealistic, and comprehensive performance benchmarking against algorithms from other domains remains challenging. Furthermore, in complex networks, the problem of finding an optimal path with minimum weight in a weighted graph aligns best with the A* algorithm, making it the most favored approach in related research. Then again, the traditional A* algorithm exhibits certain drawbacks, such as generating numerous unreasonable turns due to the neglect of UAV flight characteristics and lacking the capability to avoid obstacles. Therefore, to overcome these shortcomings, we can come up with solutions that imitate the training features of machine learning methods, integrate radio maps to construct navigation models using complex networks, and enhance the A* algorithm to endow the path selection with the capabilities of incorporating UAV flight characteristics and obstacle avoidance. This can not only make the planning effectiveness of the A* algorithm comparable to machine learning methods but also significantly reduce the model construction time, compared to machine learning methods, and additionally enhance the generalization ability of the model, providing a reference for more rapidly obtaining reasonable results for this problem.

This paper studies the path planning problem for cellular-connected UAVs in the context of a 3D radio map of a dense urban area. Firstly, a navigation model based on a complex network is constructed according to the radio map. Then, based on this navigation model, an improved A* path planning algorithm, UAVRM-A*, is proposed, which aligns with UAV flight characteristics and possesses obstacle avoidance capabilities. Experimental validation reveals that, under the same scenario, the proposed algorithm can avoid obstacles and produce the path without excessively sharp turns, making it more suitable for UAV flight characteristics compared to the traditional A* algorithm. When compared to the DRL method, the obtained results show similar flight durations but significantly reduced radio outage probability, and the modeling time of the navigation model is notably shorter than the training time required for the DRL model, proving the effectiveness of the proposed algorithm. The contributions of this paper can be summarized as follows:

1. Navigation Model Based on Complex Networks: We constructed a novel navigation model grounded in complex network theory, utilizing a 3D radio map to represent the UAV’s operational environment. This model defines the UAV’s action space and assigns path weights based on optimal values tailored to the specific path planning problem. This approach provides a robust computational framework that supports efficient and accurate pathfinding, offering a significant advancement in modeling UAV navigation in real-world environments.
2. Proposed UAVRM-A* Algorithm: Building upon the traditional A* algorithm, we propose the UAVRM-A* algorithm, which incorporates essential UAV flight characteristics and obstacle avoidance capabilities. Unlike the traditional A* method, UAVRM-A* accounts for obstacles such as buildings and ensures that flight paths respect the UAV’s turning radius, preventing abruptly or overly sharp turns. This enhancement significantly improves path smoothness and operational safety, aligning the pathfinding process with the realistic constraints of UAV flight.
3. Comparative Performance Evaluation: We conducted extensive experiments in a dense urban scenario using the generated 3D radio map, comparing the performance of the UAVRM-A* algorithm against both the traditional A* algorithm and the DRL method. The results validate the superiority of UAVRM-A* in terms of obstacle avoidance, alignment with UAV flight dynamics, and computational efficiency. This comparative analysis demonstrates that UAVRM-A* not only achieves performance on par with DRL but does so with significantly reduced computational time and radio outage duration.

The arrangement of the main content of this paper is as follows: Section 2 reviews the related work. Section 3 introduces the definition of the research problem, including the radio map used in the study and a detailed description of the problem model, outlining the research objectives. Section 4 elaborates on the proposed algorithm, including the construction of the navigation model, an introduction to the traditional A* algorithm, and a description of the proposed UAVRM-A* algorithm. Section 5 presents experimental analysis to verify the effectiveness of the proposed algorithm. Section 6 summarizes the paper and provides future outlook.

## 2. Related Work

### 2.1. Related Work About Path Planning for Cellular-Connected UAVs

Research on path planning for cellular-connected UAVs has yielded a substantial amount of findings. In existing studies, path planning algorithms are primarily categorized into those based on complex networks and those based on machine learning, with the latter being the most mainstream approach. In the realm of machine learning, research initiates with simple channel modeling based on Line-of-Sight (LoS) channels. Bulut first investigated trajectory optimization under limited connectivity conditions between UAVs and ground cellular networks, acknowledging that cellular networks and UAVs can experience interruptions and disconnections, but the interruption time must be confined within a certain threshold, thereby obtaining a highly complex solution through a linear programming approach [17]. The Walid team utilized machine learning to optimize the flight trajectory of cellular-connected UAVs in air-to-ground interference scenarios [18]. Among machine learning approaches, deep reinforcement learning (DRL) has garnered the most attention. The fundamental idea of DRL is to convert the selection of flight actions into a Markov Decision Process (MDP), utilizing UAV observations of network states to optimize real-time trajectory planning for the UAVs [19]. In terms of DRL training models, recent research has predominantly employed the Deep Q-Network (DQN) and Deep Deterministic Policy Gradient (DDPG) models [20]. Considering the inherently low training efficiency of these models, Wang leveraged priority experience replay to accelerate the training process of DRL path planning algorithm [21]. Song integrated federated learning into DRL to mitigate data isolation issues, thereby enhancing training performance [22]. Betalo et al. investigated path planning for laser-powered UAVs and proposed the Multi-Agent Deep Q-Network (MADQN) algorithm, which optimizes dynamic charging strategies and energy allocation during path planning [23]. Subsequently, they enhanced this approach by introducing a dual Deep Q-Network structure and proposing the MA-DDQN algorithm, further improving energy consumption and mission completion time [24]. The aforementioned studies all assume that UAVs operate within a plane at a constant altitude. Gesbert et al. extended the scenario to 3D spaces, utilizing a 3D map for the trajectory optimization of cellular-connected UAVs while considering network connectivity constraints [25]. The Zeng team introduced a radio map for the first time, proposing an integrated communication and navigation trajectory optimization strategy based on DRL [26]. Hao et al. reconstructed radio maps using a deep image prior mechanism, improving the DRL algorithm and ultimately reducing the flight energy consumption of UAVs [27].

In the realm of complex networks, which are complexity systems grounded in graph theory, research initially commenced with classical graph theoretic methodologies. Traditional graph theory-based path planning algorithms include Dijkstra, Floyd, A*, Ant Colony Optimization (ACO), and Rapidly exploring Random Tree (RRT) [28,29]. However, these traditional algorithms evidently cannot fully meet the flight requirements of UAVs, prompting related research to focus on improving them. Among path planning algorithms for discretized spaces, the A* algorithm is the most favored. The A* algorithm, improved by Ju et al., possesses the characteristic of enabling UAVs to avoid obstacles while also addressing the issue of traditional algorithms failing to provide optimal solutions under specific conditions [30]. Li et al. tackled the problem of excessively sharp turning points in generated paths of A*, which are incompatible with UAV characteristics, and applied curve smoothing to the paths [31]. Zhang et al. integrated regional connection calculus theory, achieving efficient and stable performance in complex UAV path planning scenarios [32]. However, these studies still rely on idealized Euclidean distances as weight settings and overly simplify the structure and scale of their scenarios. In contrast, Bai et al. addressed the collaborative delivery problem involving high-capacity UAVs and trucks, set weights according to the characteristics of the problem, and designed several decoupled heuristic algorithms [33]. In the path planning scenario involving nonholonomic constraints and multi-dimensional state spaces, the RRT algorithm has garnered the most attention. Gu et al. improved the RRT algorithm to reduce the randomness of sampling and enabled UAVs to avoid obstacles through collision detection [34]. Zhao et al., in combination with the artificial potential field method, proposed the APF-RRT* algorithm, enhancing sampling efficiency and convergence speed [35]. The studies mentioned above primarily consider the conventional flight characteristics of UAVs without taking into account the need for networked UAVs to maintain stable network connectivity. Zhang et al. investigated the trajectory optimization strategy based on graph theory to ensure connectivity between UAVs and ground cellular networks while maintaining cellular connectivity [36]. Yang proposed a low-complexity trajectory optimization scheme under the same circumstances [37]. Chen et al. leveraged radio maps to design a graph theoretic path optimization method constrained by communication quality, which was applied to a cargo delivery system [38]. In addition to the aforementioned aspects, there are also studies in other domains. For instance, Carrese et al. employed a mixed-integer programming model and utilized heuristic methods to optimize the routing and flight scheduling of a fleet of UAVs tasked with monitoring vehicle status in a shared mobility environment [39]. Chu et al. discussed the application of a fast heuristic algorithm based on particle swarm optimization in UAV path planning and offered an example of applying metaheuristic methods to UAV-related problems [40].

### 2.2. Common Issues of Related Work

With an overview of the research above, it can be found that numerous common issues still persist in the study of path planning for networked UAVs. Firstly, most studies exhibit deficiencies in the setup of application scenarios, primarily manifested in the following aspects:

- Overly Idealized Scenarios: Path planning solutions in the majority of studies solely consider the factor of the shortest path from the starting point to the destination, without incorporating constraints from real-world environments, such as maintaining network coverage along the generated path and avoiding obstacles.
- Oversimplified Experimental Environments: These studies predominantly employ 2D environments as their experimental settings, assuming that UAVs fly at a constant altitude, while research on path planning in 3D environments remains limited. In addition, the experimental scenarios are overly simplistic; for instance, in studies applied to urban areas, the city models used in experiments often exhibit overly regular or sparse building distributions, significantly differing from real-world environments.

Secondly, in terms of the algorithms employed, machine learning approaches, particularly deep reinforcement learning, serve as the most prevalent methods. These methods can swiftly and dynamically derive path results upon the completion of training and exhibit excellent performance. However, relevant research merely discusses the performance of path planning to demonstrate the superiority of the proposed methods while ignoring some common drawbacks of machine learning in the model training process:

- Lengthy training time: To achieve satisfactory results, machine learning typically requires numerous iterations in the training process to ensure model convergence, with each training session needing to commence from scratch. This results in significantly prolonged training time.
- Complex structure of training samples: To ensure effective training outcomes, machine learning generally necessitates a sufficient quantity of samples and adequately comprehensive parameters.
- Low generalization ability of the trained model: The models obtained after training in research are only applicable to specific environments or scenarios. In reality, environments are constantly changing. Once an environmental change occurs, the models become ineffective, necessitating substantial time to retrain for the new environment and obtain a new model.

Furthermore, regarding complex network methodologies, their advantages lie in their short modeling time and strong adaptability of the models to environmental changes. However, relevant research falls short in the setup of experimental environments, which hinders the manifestation of these advantages. This is specifically reflected in the following aspects:

- Overly Idealized Experimental Settings: Complex network methodologies typically reconstruct the experimental environment into a weighted graph serving as navigation model, seeking an optimal path with the optimal weight. Yet relevant studies generally lack detailed discussions on the navigation model and overly simplify the structural settings within the model. The weights assigned to each path still adhere to idealized scenarios or are arbitrarily set without incorporating factors related to the actual environment, resulting in a mismatch with the form of the research problem.
- Limitations of Comparative Studies: Relevant research primarily focuses on improving traditional graph theory algorithms; hence, the scope of comparative studies is limited to algorithms within the field of graph theory. Although there is no inherent restriction preventing the inclusion of algorithms from other fields in comparative studies, the diverse solution forms and applicable environments across various fields pose significant challenges in finding a single environment that can accommodate all these algorithms simultaneously. Consequently, few studies have taken multiple-field algorithm comparison into consideration.

## 3. Problem Definition

### 3.1. Radio Map Model

A radio map indicates the radio conditions at various locations on the map, providing a basis for networked UAVs to ensure network connectivity. This paper adopted the radio map that estimates radio outage probability based on the signal-to-interference noise ratio (SINR) in a dense urban environment [26]. The map covers a $D\times D{\;m}^{2}$ area of a dense urban region, with *N* base stations and a total of *M* cells, where the antenna model design follows 3GPP standards [41]. The radio map is constructed based on radio measurement data from UAVs. Assume the UAV is located at position $u_{t}$ at time *t*, and the instantaneous signal power measured from cell m∈{1,…,M} is p(t,m). The cell currently engaged in radio communication with the UAV is designated as c(t). Then, the SINR at time *t* can be expressed as

(1) $$\gamma (t)=\frac{p(t,c(t))}{\sum_{m\neq c(t)}p(t,m)}.$$

For a given location, signal power experiences both large-scale fading and small-scale fading. Large-scale fading is determined by the positions of the UAVs, the base stations, and the buildings between them. Since the positions of base stations and buildings are fixed, it can be assumed that for the same location, large-scale fading remains constant over time. In contrast, small-scale fading is a small enough random variable. Therefore, the signal power at time t is equivalent to the instantaneous signal power from the connected cell c(t) at location $u_{t}$, with the presence of small-scale fading $\tilde{h}(c(t))$. Similarly, γ(t) is also a random variable that depends on $u_{t}$, c(t), and $\tilde{h}(c(t))$. Consequently, Formula (1) can be transformed as follows:

(2) $$\begin{array}{l} \gamma (t)=\gamma (u_{t},c(t),\tilde{h}(c(t)))\\ =\frac{p(u_{t},c(t),\tilde{h}(c(t)))}{\sum_{m\neq c(t)}p(u_{t},m,\tilde{h}(m))}. \end{array}$$

Given that the radio connection is deemed interrupted when the SINR falls below a certain threshold, that is, $\gamma (t)<\gamma_{th}$, the outage probability can be defined as

(3) $$P_{out}(u_{t},c(t))=\mathrm{Pr}\{\gamma (t)<\gamma_{th}\}$$

where Pr{·} represents the probability of an event occurring. Recognizing that the UAV does not rely on a single measurement but rather conducts multiple consecutive measurements within a very short time interval, when the number of measurements is sufficiently large, the expected outage probability approaches the true outage probability. Therefore, the expected outage probability for each cell can be obtained through mathematical expectation, upon which the decision to connect to cell c(t) is based. We define the outage indicator function $I(u,m;\tilde{h})$ as

(4) $$\begin{array}{l} I(u_{t},m,\tilde{h}(m))=\left\{\begin{array}{l} 1,\;\gamma (u_{t},m,\tilde{h}(m))<\gamma_{th}\\ 0,\;\gamma (u_{t},m,\tilde{h}(m))\geq \gamma_{th} \end{array}\right.,\\ m\in \{1,\ldots ,M\}. \end{array}$$

Assuming that the UAV, located at position $u_{t}$ at time *t*, performs *J* measurements for each cell, with $\tilde{h}(m,j)$ representing the small-scale fading for the *j*-th measurement of cell *m*, the expected outage probability can be derived as

(5) $$\begin{array}{ll} {\bar{P}}_{out}(u_{t},m) & =\mathrm{Pr}\{\gamma (u_{t},m;\tilde{h}(m))<\gamma_{th}\}\\ & =Ε\left(\tilde{h}\right)\\ & =\frac{1}{J}\sum_{j=1}^{J}I(u_{t},m,\tilde{h}(m,j)). \end{array}$$

Consequently, the UAV at time *t* selects the sector with the minimum outage probability for connection, that is, $c(t)=\arg\;\underset{m\in \{1,\ldots ,M\}}{\min}{\bar{P}}_{out}(u_{t},m)$. Thus, the outage probability at position $u_{t}$ in the radio map is expressed as

(6) $${\hat{P}}_{out}(u_{t})={\bar{P}}_{out}(u_{t},c(t))=\underset{m\in \{1,\ldots ,M\}}{\min}{\bar{P}}_{out}(u_{t},m).$$

It is essential to note that, as inferred from the representation and storage methods of the graph, a radio map is not a continuous map. Instead, it is constructed as a discrete spatial representation based on an urban model, partitioned at a specified granularity. Evidently, employing a finer granularity enables the radio map to indicate radio information locations with greater precision within the same coverage area. However, this simultaneously leads to a substantial increase in the number of locations requiring radio measurements, eventually resulting in a significant escalation of the measurement and computational costs associated with map construction. Therefore, in practice, the granularity should be determined based on the requirements of the path planning task, and the radio map should be constructed on-demand to match this granularity. Additionally, considering the practical difficulty of real-world UAVs in precisely controlling their positions on grid points during radio measurements, coupled with the inherent impossibility of performing infinite measurements, a radio map cannot be constructed solely from measured data. The radio map methodology adopted in this paper allows for the utilization of supervised learning to learn from limited measurement data. It leverages interpolation-based estimation to predict radio information at unmeasured locations, thereby complementing the whole radio map, and continuously validating and calibrating through measurements and learning processes [26].

### 3.2. Problem Model

To elaborate on the specific scenarios and objectives of the research, it is necessary to construct a problem model. Based on the known radio map, this paper considers the scenario of cellular-connected UAV performing flight missions in dense urban area, with the objective of finding a path from starting point $u_{I}$ to destination point $u_{F}$ within the area. This path is characterized by

(1) Having the shortest flight time *T*;
(2) Experiencing the minimal radio outage duration depending on the outage probability ${\hat{P}}_{out}$.

**Figure 2:**
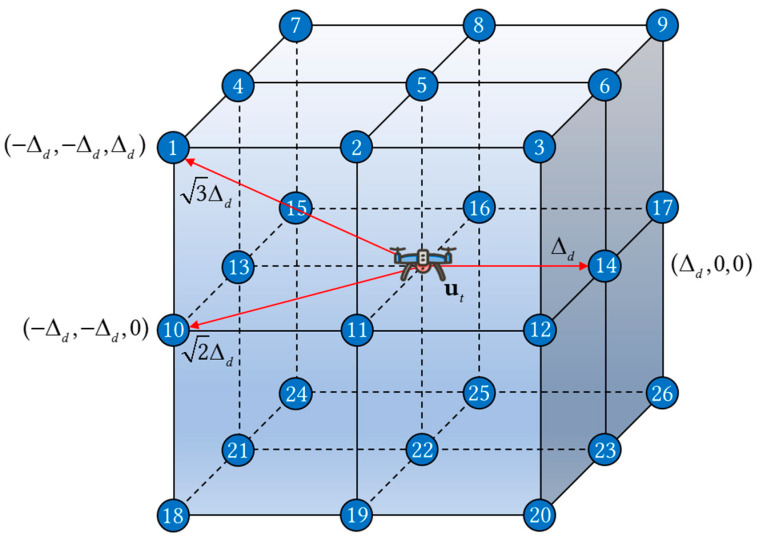
The 26 directions in the action set.

Assuming that the operational range during the flight mission is a $D\times D{\;m}^{2}$ area and the flying speed is *V*, the aforementioned problem can be formulated into the following problem model:

(7) $$\begin{array}{ll} P:\;\underset{T,\left\{u_{t}\right\}}{\min}T+\int_{0}^{T}{\hat{P}}_{out}(u_{t})dt & \left(a\right)\\ s.t.\;u_{0}=u_{I}=\left(x_{I},y_{I},z_{I}\right), & \left(b\right)\\ \;\;\;\;u_{T}=u_{F}=\left(x_{F},y_{F},z_{F}\right), & \left(c\right)\\ \;\;\;\;\left\|{\dot{u}}_{t}\right\|=V, & \left(d\right)\\ \;\;\;\;0\leq x_{t}\leq D,\;0\leq y_{t}\leq D,\;z_{\min}\leq z_{t}\leq z_{\max},\forall t\in \left[0,T\right] & \left(e\right) \end{array}$$

where (a) is the objective function, (b) and (c) contain information about the starting and destination points, as well as the coordinate representation in a 3D space, (d) indicates the existing speed constraint during decision process, and (e) describes the constraints on the operational range. However, due to the non-convexity of this problem model and the continuous action space, direct solution would lead to a rapid increase in complexity, resulting in the curse of dimensionality. Given that the three-dimensional path navigation problem can be represented in the form of a graph, and considering that the UAVs operated in dense urban environments are predominantly quadrotor UAVs, which exhibit discrete motion characteristics, a complex network can be employed to transform the path navigation problem into a discrete path planning problem among three-dimensional grid nodes for solution. By utilizing a complex network model, the action space is divided into a 3D Cartesian coordinate system grid with a granularity of $\Delta_{d}$, where each node on the grid represents a location that the UAV can reach (including the starting and destination point). In a single action, the UAV flies from one node to an adjacent node (including diagonal moves), with a maximum movement distance of $\sqrt{3}\Delta_{d}$. Consequently, the action can be executed in up to 26 different directions, collectively referred to as action set A, as shown in Figure 2:

(8) $$A=\left\{\begin{array}{l} (\Delta_{d},0,0),(\Delta_{d},\Delta_{d},0),(\Delta_{d},-\Delta_{d},0),\\ (\Delta_{d},0,\Delta_{d}),(\Delta_{d},\Delta_{d},\Delta_{d}),(\Delta_{d},-\Delta_{d},\Delta_{d}),\\ (\Delta_{d},0,-\Delta_{d}),(\Delta_{d},\Delta_{d},-\Delta_{d}),(\Delta_{d},-\Delta_{d},-\Delta_{d}),\\ (0,\Delta_{d},0),(\emptyset ),(0,-\Delta_{d},0),\\ (0,\Delta_{d},\Delta_{d}),(0,0,\Delta_{d}),(0,-\Delta_{d},\Delta_{d}),\\ (0,\Delta_{d},-\Delta_{d}),(0,0,-\Delta_{d}),(0,-\Delta_{d},-\Delta_{d}),\\ (-\Delta_{d},0,0),(-\Delta_{d},\Delta_{d},0),(-\Delta_{d},-\Delta_{d},0),\\ (-\Delta_{d},0,\Delta_{d}),(-\Delta_{d},\Delta_{d},\Delta_{d}),(-\Delta_{d},-\Delta_{d},\Delta_{d}),\\ \left(-\Delta_{d},0,-\Delta_{d}),(-\Delta_{d},\Delta_{d},-\Delta_{d}),(-\Delta_{d},-\Delta_{d},-\Delta_{d}\right) \end{array}\right\}$$

Obviously, when $\Delta_{d}$ is small enough, minimizing the flight time *T* in the problem objective is equivalent to minimizing the total distance of all the actions performed by the UAV during the flight. However, as $\Delta_{d}$ approaches 0, the action space tends to be continuous. Considering that the problem complexity is approximately on the order of $O(n^{3})$, the excessive trend towards a continuous space rapidly increases the problem complexity, resulting in reducing the pathfinding efficiency of the algorithm. Therefore, $\Delta_{d}$ cannot be arbitrarily assigned; rather, in practice, it should be a reasonable value depending on the maneuverability of the UAV, with the premise of ensuring the establishment of the aforementioned equivalence condition. In summary, the above problem model can be transformed into the following form:

(9) $$\begin{array}{ll} P^{\prime }:\;\underset{N,\left\{u_{n}\right\}}{\min}\sum_{n=0}^{N-1}\left(\left\|u_{n+1}-u_{n}\right\|+{\hat{P}}_{out}(u_{n+1})\right) & \left(a\right)\\ s.t.\;u_{0}=u_{I}=\left(x_{I},y_{I},z_{I}\right), & \left(b\right)\\ \;\;\;\;u_{N}=u_{F}=\left(x_{F},y_{F},z_{F}\right), & \left(c\right)\\ \;\;\;\;u_{n+1}-u_{n}\in A,\;\left\|u_{n+1}-u_{n}\right\|\leq \sqrt{3}\Delta_{d}, & \left(d\right)\\ \;\;\;\;0\leq x_{n}\leq D,\;0\leq y_{n}\leq D,\;z_{\min}\leq z_{n}\leq z_{\max},\forall n\in \left[0,N\right] & \left(e\right) \end{array}$$

where *N* is the total steps of motions in the flying task.

## 4. Methodology

**Figure 3:**
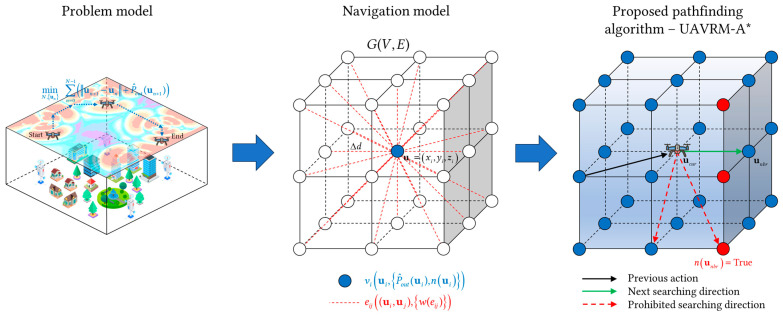
Methodology for the problem model.

After the problem model is established, a path planning algorithm is required to address the issue. Generally, for a graph-theoretic algorithm, the factors influencing the effectiveness and accuracy of the algorithm are not solely related to the algorithm mechanism; the assignment of path weights is equally crucial. However, existing research has predominantly focused on refining the algorithm mechanism, while the assignment of weights has been rather arbitrary, resulting in a limited practical applicability of the algorithms. Furthermore, when the aforementioned problem is discretized using a complex network model, the action space is divided into a grid-like shape. Within such an action space, the form of solving path planning is evidently most compatible with the A* algorithm. Conversely, the algorithms that tend towards continuous spaces, such as RRT* (which employs random sampling) and Hybrid-A* (which merely discretizes actions while maintaining a continuous representation of the state space), exhibit poor compatibility with discrete spaces. Yet, the traditional A* algorithm solely considers the factor of optimal weight and lacks the capability to take into account other constraints during pathfinding. Therefore, this paper has come up with a methodology for the problem model, as illustrated in Figure 3. Initially, a navigation model is constructed based on the problem model, ensuring that the weight assignment aligns with the requirement of the problem. Subsequently, according to the navigation model, the traditional A* is adopted as the fundamental pathfinding algorithm and integrated with the scenario of UAV path planning in a radio map environment, leading to the proposal of an improved A* algorithm—UAVRM-A*.

### 4.1. Navigation Model

Typically, a map model serves as a static representation of physical/virtual space and is not responsible for defining the action space or assigning edge weights required by the algorithm. In constructing a navigation model, the objective is to reconfigure an actual map scenario into a form that can be solved by the path planning algorithm, while also recording the weights of various paths to provide a pathfinding environment for the algorithm. However, a navigation model is not a generic or readily available solution. In practical applications, it constitutes a highly customized tool, requiring specific design based on the problem definition and the algorithm. Consequently, the resulting navigation model is generally applicable only to path planning problems involving agents with analogous behavioral patterns in similar environments. Furthermore, its design is typically tailored to specified algorithms compatible with the problem-solving formulation, rendering it ill-suited for accommodating other approaches. Considering that path planning inherently involves direction and the need to find the path with optimal weight, a directed weighted attributed graph can be employed to build the navigation model. This paper will refer to the navigation model construction method proposed in existing research and integrate it with the problem model to construct a navigation model [42].

Given that path planning inherently involves directionality and necessitates the searching of paths with optimal weights, a directed weighted attributed graph can be adopted to construct the navigation model. In complex network theory, a directed weighted attributed graph is represented as G(V(AV,LV),E(AE,LE)). Here, V(AV,LV) represents the set of all nodes $v_{i}(a_{i},l_{i})$, and E(AE,LE) denotes the set of all edges $e_{ij}(a_{ij},l_{ij})$. Detailed specifications of the elements within these sets are provided in Table 1.

According to the properties of the directed weighted attribute graph mentioned above, the information in the problem model is incorporated into the graph to construct the navigation model. For nodes, as described in Section 3.2, the nodes on the grid divided by the action space are set as the nodes of the navigation model; then, a node can be represented as

(10) $$\begin{array}{l} v(u_{i})=v_{i}\left(a_{i},l_{i}\right),\\ a_{i}=u_{i},\\ l_{i}=\left\{\mathrm{outage}={\hat{P}}_{out}(u_{i})\right\},\\ u_{i}=(x_{i},y_{i},z_{i}). \end{array}$$

Here, the coordinate of the position is set as the identifier of the node. In the attribute set, the outage probability of the position is added as the “outage” attribute of the node.

For edges, directed edges are added between adjacent nodes according to each action in action set A, and their weights are placed in the attribute set. Therefore, they are represented as

(11) $$\begin{array}{l} e(u_{i},u_{j})=e_{ij}\left(a_{ij},l_{ij}\right),\\ a_{ij}=(u_{i},u_{j}),\\ l_{ij}=\left\{\mathrm{weight}=w(e(u_{i},u_{j}))\right\},\\ u_{j}-u_{i}\in A. \end{array}$$

Next is the discussion about the setting of edge weights. Referring to the existing reinforcement learning approach [43], the training process typically involves transforming the problem model into a Markov decision process and conducting exploration with a “reward-return” mechanism. Through repeated exploration, the model continuously learns to find a path with optimal return. However, this approach usually requires a sufficiently large number of explorations to ensure the convergence of the training model, leading to the issue of an excessively long training time cost. Nevertheless, it is evident that the setting of return values during exploration is highly consistent with the form of the optimal value required by the problem model. Therefore, the edge weight is set to be consistent with the form of the numerical value required by the problem model, specifically

(12) $$w(e(u_{i},u_{j}))=\lambda_{1}\left\|u_{j}-u_{i}\right\|+\lambda_{2}{\hat{P}}_{out}(u_{j})$$

where $\lambda_{1},\lambda_{2}$ are weight parameters that need to be set according to the specific environment.

In conclusion, the construction method of a UAV navigation model under radio map environments based on complex networks can be summarized into the following steps:

**Input:** Radio map.

- **Step 1:** Construct the graph G(V(AV,LV),E(AE,LE)).
- **Step 2:** Obtain each location $u_{i}$ based on the problem model in Formula (9) and retrieve its outage probability ${\hat{P}}_{out}(u_{i})$ from the radio map. Then, add node $v(u_{i})$ to *V* according to Formula (10).
- **Step 3:** Following the actions in the action set, add a directed edge $e(u_{i},u_{j})$ between all adjacent nodes $v(u_{i})$ and $v(u_{j})$ to *E* based on Formula (11). Calculate the edge weight $w(e(u_{i},u_{j}))$ and store it in the “weight” attribute according to Formula (12) using the “outage” attribute of the corresponding nodes.

**Output:** Navigation model *G*.

Furthermore, it should be noted that this model possesses significant generalization advantages compared to models derived from machine learning training. As understood from the characteristics of machine learning training, its iterative exploration based on a “reward-return” mechanism is typically conducted within the same environment towards a fixed destination. This process results in a model that develops a specific “memory” for this particular goal after training. Once the environment or destination changes, so does the goal, and the “memory” will no longer be valid. Consequently, the model must be retrained to acquire new “memory” generating an entirely new model. In contrast, the navigation model, as a model grounded in graph theory, does not rely on memorizing specific data patterns. Instead, it depends on an inherent “understanding” of universal spatial relationships and optimization principles. Barring scenarios involving drastic or entirely new environmental changes necessitating model reconstruction, the model structure itself can simply be updated (e.g., by refreshing node or edge attributes). The underlying algorithm logic remains entirely unchanged and can operate on the updated model to perform pathfinding. Critically, it can accept arbitrary start and destination points as algorithm inputs. This demonstrates that the navigation model exhibits superior adaptability to changes in both environment and destination compared to machine learning-trained models. This generalization advantage is an intrinsic outcome of its foundation in graph theory and logical search principles.

### 4.2. Traditional A* Algorithm

The navigation model is not constructed within the pathfinding algorithm itself. Instead, it is pre-built and subsequently provided as input data for the algorithm to read. Consequently, after constructing the navigation model, a suitable pathfinding algorithm must be selected. Considering that the constructed navigation model has a discrete grid structure, and each action moves to an adjacent node in the grid, such movement characteristics are highly compatible with A* and its related algorithms, which are designed for discrete graph search. Among these, the traditional A* algorithm is typically the preferred option and the foundational paradigm for solving this problem, as it guarantees finding the weight-optimal path. Therefore, this paper adopts the traditional A* algorithm as the fundamental pathfinding algorithm for the navigation model.

The A* algorithm is a kind of static global path planning algorithm that contains a heuristic searching process. Compared to Dijkstra, A* incorporates heuristic estimation to accelerate the pathfinding process, reducing the number of search nodes and thereby increasing the pathfinding efficiency. At state *n* of pathfinding, it evaluates the current state by the following evaluation function:

(13) f(n)=g(n)+h(n)

where g(n) is the total cost from the starting point to state *n*, and h(n) is the estimated cost from state *n* to the destination point. When incorporating this function into the navigation model, state *n* corresponds to its position $u_{n}$. Evidently, g(n) equals the accumulated weight up to reaching that state, and h(n) is conventionally assumed in the traditional A* algorithm to be equivalent to the Euclidean distance from state *n* to the goal. Consequently, within the navigation model, the evaluation function is formulated as

(14) $$\begin{array}{c} f(u_{n})=g(u_{n})+h(u_{n}),\\ g(u_{n})=\sum_{i=0}^{n-1}w(e(u_{i},u_{i+1})),\\ h(u_{n})=\left\|u_{n}-u_{F}\right\|. \end{array}$$

### 4.3. UAVRM-A* Algorithm

In the context of the problem model, the traditional A* algorithm can theoretically provide an optimal path that guarantees favorable radio conditions. However, practical considerations reveal certain impracticalities in such a path. Firstly, the path may contain sharp turns that overlook the unique flight characteristics of UAVs. Secondly, the designated role of the traditional A* algorithm is solely to perform pathfinding utilizing a heuristic function. It lacks explicit, inherent capabilities for obstacle recognition and avoidance. In reality, depending on the tasks performed by the networked UAVs, they may fly in low-altitude airspace (approximately 20 m to 60 m above the ground), where buildings are abundant in dense urban areas, necessitating obstacle avoidance. Therefore, modifications to the traditional A* algorithm are necessary to impose relevant constraints during pathfinding, thus generating more practical optimal paths. This paper integrated the traditional A* algorithm with UAV path planning scenarios in the radio map environment, proposing an improved A* algorithm—the UAVRM-A* algorithm.

For obstacle occlusion recognition, considering that the responsibility for marking obstacles resides with the navigation model, while the traditional A* algorithm treats the navigation model solely as input data, it possesses neither the responsibility nor the inherent capability to perform further modifications or markings within the navigation model. Indeed, it lacks even the responsibility to actively assess node traversability. Therefore, the improvement should adopt a dual approach involving both the navigation model and the algorithm pathfinding mechanism. Initially, an attribute can be added to each node in the navigation model to determine whether the position lies within a no-fly zone. To simplify the research, only no-fly zones caused by static obstacles like buildings are considered. Thus, Equation (10) will be transformed into the following form:

(15) $$\begin{array}{l} v(u_{i})=v_{i}\left(a_{i},l_{i}\right),\\ a_{i}=u_{i},\\ l_{i}=\left\{\mathrm{outage}={\hat{P}}_{out}(u_{i}),\mathrm{no}\text{\_}\mathrm{fly}=n\left(u_{i}\right)\right\},\\ u_{i}=(x_{i},y_{i},z_{i}). \end{array}$$

Here, a Boolean attribute “no_fly” is added to identify whether the node is located in a no-fly zone, in order to meet the path planning requirements under scenarios where obstacles exist in the area, such as building occlusion. Regarding the judgment of the “no_fly” attribute, when constructing the navigation model, the urban model is incorporated as an additional input to obtain the information data about obstacles. Then, when adding nodes, the model automatically determines whether the node is located within an obstacle to set the “no_fly” attribute accordingly. Subsequently, during the A* algorithm pathfinding process, a constraint is imposed: when the current position is $u_{cur}$, if $n(u_{nbr})=\mathrm{True}$ (indicating the neighbor node $u_{nbr}$ is in a no-fly zone), the node is skipped, thereby preventing the UAV from entering the range of the obstacle.

To combine the flight characteristics of UAVs, considering that UAVs flying at high speeds with the 26 actions in action set A struggle to execute turns exceeding right angles, constraints on the directions can be added to avoid sharp turns in the path, leading to a more realistic path.

When searching for a path, assuming the current state is located at position $u_{cur}$, when action $\delta_{cur}$ reaches this position and the next neighboring node being searched for is $u_{nbr}$, then, the action to move to this neighboring node is $\delta_{nbr}=u_{nbr}-u_{cur}$. Thus, the turning angle to move to $u_{nbr}$ can be calculated as

(16) $$\theta =\arccos\left(\frac{\delta_{nbr}\cdot \delta_{cur}}{\left|\delta_{nbr}\right|\left|\delta_{cur}\right|}\right)$$

In particular, when starting the pathfinding from the starting point, that is, when $u_{cur}=u_{I}$, $\delta_{cur}=0$. The UAV only moves along the direction of the first action executed without generating a turning angle; hence, at this point, it is defined as

(17) $$\cos\theta_{I}=\frac{\delta_{nbr}^{2}}{{\left|\delta_{nbr}\right|}^{2}}=1\;\Rightarrow \;\theta_{I}=0$$

From this, during the pathfinding process in the A* algorithm, a constraint is added. When searching for neighboring nodes, turning angle θ is calculated. If this turning angle reaches or exceeds a right angle ($\theta \geq \frac{\pi }{2}$), the node is skipped to limit the turning angle of the UAV.

In summary, the steps of the UAVRM-A* algorithm can be summarized as follows:

**Input:** Navigation model *G*, starting point $u_{I}$, destination point $u_{F}$.

- **Step 1: Initialization.** Choose the starting point node $u_{I}$ from the navigation model and place it into the open list **OPEN**. Calculate $h(u_{I})$ based on Formula (14); set $g(u_{I})\leftarrow 0$, $f(u_{I})\leftarrow h(u_{I})$, $\delta_{I}=0$.
- **Step 2: Selection.** Select the node with the minimum $f(u_{n})$ value from the open list as the current node, denoted as $u_{cur}$. Remove $u_{cur}$ from the open list **OPEN** and add it to the closed list **CLOSE** to avoid duplicate visits.
- **Step 3: Expand Nodes.** Identify the neighbor nodes based on all directed edges originating from $u_{cur}$. For each neighbor node $u_{nbr}$ of $u_{cur}$,
  (a) Calculate $g_{cur}(u_{cur})$ according to Formula (14).
  (b) If $u_{nbr}\notin OPEN\wedge u_{nbr}\notin CLOSE$, it indicates that $u_{nbr}$ has not been explored. Add $u_{nbr}$ to **OPEN** and set $u_{nbr}.parent\leftarrow u_{cur}$, $g(u_{nbr})\leftarrow g_{cur}(u_{nbr})$, $\delta_{nbr}\leftarrow u_{nbr}-u_{cur}$. Calculate $h(u_{nbr})$ and $f(u_{nbr})$ based on Formula (14). Meanwhile, if $n(u_{nbr})=\mathrm{True}$, add it to **CLOSE** instead of **OPEN** to prevent obstacle collision.
  (c) If $u_{nbr}\in OPEN$, calculate turning angle θ according to Formulas (16) and (17). Then if $\theta <\frac{\pi }{2}\;\wedge \;g_{cur}(u_{cur})<g(u_{cur})$, a valid and more optimal path is found. Update the parent by $u_{nbr}.parent\leftarrow u_{cur}$, $g(u_{nbr})\leftarrow g_{cur}(u_{nbr})$, $\delta_{nbr}\leftarrow u_{nbr}-u_{cur}$; recalculate $h(u_{nbr})$ and $f(u_{nbr})$. Otherwise, do nothing.
  (d) If $u_{nbr}$ does not meet the above conditions or $u_{nbr}\in CLOSE$, do nothing.
- **Step 4: Loop.** Repeat Step 2 and Step 3 until $u_{cur}=u_{F}$ or OPEN=∅.
- **Step 5: Return Result.** When $u_{cur}=u_{F}$, the destination is reached. Backtrack from the destination to the starting node using the parent of each node to generate the optimal path $\left\{u_{0},\ldots ,u_{N-1},u_{N}\}=\{u_{I},\ldots ,u_{F}.parent,u_{F}\right\}$. When OPEN=∅ and no path to the destination is found, it indicates that there is no path from the start point to the destination.

**Output:** Optimal path sequence $\left\{u_{0},\ldots ,u_{N-1},u_{N}\right\}$. When no path is found, it is ∅.

## 5. Evaluation

### 5.1. Evaluation Environment

**Figure 4:**
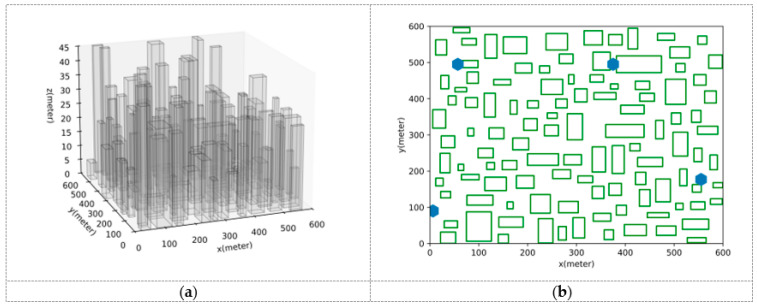
The urban scenario adopted as the evaluation environment; (**a**) 3D view; (**b**) 2D planform, where green rectangles represent buildings, blue hexagons represent base stations.

**Figure 5:**
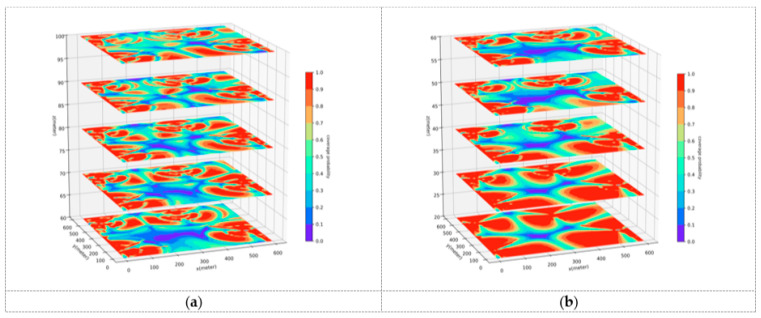
Radio map of the urban scenario. (**a**) Medium aerial area. (**b**) Low aerial area.

This part will conduct a comparative analysis of the path planning results generated by the UAVRM-A* algorithm proposed in this paper, the traditional A* algorithm, and the DRL model in the same environment to verify the effectiveness of the proposed algorithm. To ensure the representativeness of the comparative experiments, this paper refers to the method of constructing urban scenarios and radio maps in ref. [43], considering the simulated dense urban scenario shown in Figure 4 as the verification environment. The urban scenario scale D=600 m, and the maximum height of buildings $h_{bd}=45\;m$. Within this scenario, the airspace is partitioned into two distinct occasions to delineate the operational boundaries for UAVs: a medium aerial area ($z_{\min}=60\;m$, $z_{\max}=100\;m$) and a low aerial area ($z_{\min}=20\;m$, $z_{\max}=60\;m$). Separate radio maps are constructed for each of these areas, as depicted in Figure 5, with the signal coverage probability ${\hat{P}}_{cov}(u_{t})=1-{\hat{P}}_{out}(u_{t})$ indicated in conjunction with the action space granularity $\Delta_{d}=10\;m$. The underlying principles governing this approach are detailed in Section 3.1. To ensure uniformity in the verification environment, other detailed parameters of the urban scenario, the base station antenna, and the radio map are set to be the same as those used in ref. [43].

In order to ensure a reasonable, rigorous, and impartial comparative analysis, path planning algorithms were selected based on the following criteria: comparability, consistent problem objectives, strict applicability to the aforementioned scenario and its corresponding radio map, and compatibility with a unified evaluation standard. Consequently, five comparative methods were ultimately identified. Primarily, the key algorithms involved in the comparative experiments are summarized as follows:

(1) **D3QN.** The multi-step dueling Double Deep Q-Network (DDQN), which is a state-of-the-art and representative improved algorithm of DDQN in the field of DRL.
(2) Traditional A*. This is introduced in Section 4.2. This algorithm will utilize the navigation model constructed in Section 4.1 for pathfinding.
(3) UAVRM-A*. The improved A*-based algorithm proposed in this paper. The principle has been introduced in Section 4.3.

Additionally, to demonstrate a comparison with algorithms typically employed in idealized or simplified (e.g., planar) scenarios, the following benchmark methods are included for comparison:

(4) **2D.** This is the valid theoretically optimal path that can be achieved when flying at a constant height. This approach necessitates specific conditions or assumptions.
(5) **Direct.** This is the valid theoretically optimal path under idealized conditions, disregarding radio outage durations and utilizing only the Euclidean distance as the edge weight, serving as the most ideal scenario in path planning.

To uphold the fairness of the comparative validation, the settings of weight parameters for the edges in the navigation model should align with those used in the training parameters of the D3QN method. Accordingly, set $\lambda_{1}=0.1,\;\lambda_{2}=50$. The granularity of these models is consistent with that of the radio map.

Regarding the evaluation metrics employed in the experiments, to assess algorithm performance, a combination of flight time and outage time was used based on the problem model for comprehensive evaluation. To evaluate obstacle avoidance capability and the integration of UAV characteristics, the number of obstacle collisions and sharp turns encountered along the path are counted. To evaluate modeling efficiency, the modeling time is measured, with the pathfinding time provided as a supplementary reference. Given the inherent randomness of small-scale fading in the radio environment, which induces minor fluctuations in the radio map, each occasion was subjected to 20 experimental runs, and the average values of the aforementioned metrics were subsequently calculated.

The simulation environment was Python 3.12, with NetworkX toolkit employed for complex network modeling, and the neural network model of D3QN was implemented using Tensorflow 2.17. The computational hardware was 11th Gen. Core i7 CPU.

### 5.2. Evaluation Result

**Figure 6:**
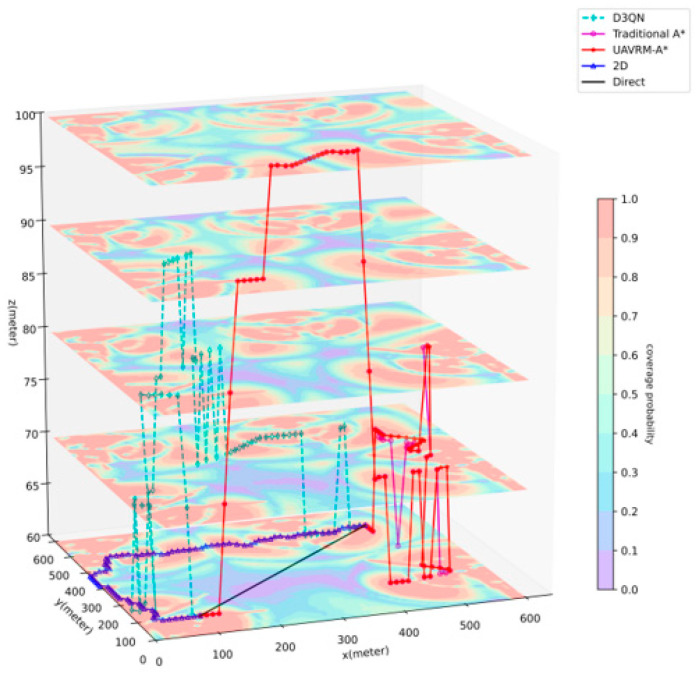
Result of solution for medium aerial area.

Figure 6 shows the path planning results of the various methods for the medium aerial area, where $z_{\min}=60\;m$, $z_{\max}=100\;m$, $u_{I}=(100,100,60)$, $u_{F}=(500,500,60)$. Table 2 lists the detailed data of the comparison results. Since $h_{bd}=45\;m<z_{\min}$, there is no need to consider the obstruction of buildings or no-fly zones within the flight area on this occasion. As evident from the results, in the benchmarks, the path of the Direct method is inevitably a straight line from the starting point to the destination, which is obviously the shortest. Then again, this straight line traverses a vast area with weak radio signal coverage, leading to a significantly long outage time. The 2D method opts for a longer route to avoid areas with weaker radio coverage within the same height plane, minimizing the outage time while significantly lengthening the path. In contrast, the primary comparison algorithms have a broader pathfinding space compared to the benchmarks, enabling them to find optimal paths by navigating to other altitudes. The optimal path found by D3QN boasts the shortest flight time among all methods, with good radio coverage, demonstrating the superiority of DRL methods. However, considering that it has poor model generalization, all 20 experimental results follow the same path; the inability to consider the flight characteristics of UAVs results in a higher number of unreasonably sharp turns in the path (an average of 10 times on this occasion). Compared to D3QN, traditional A* increases the flight time slightly but significantly reduces the interruption time. Nevertheless, based on the drawbacks of traditional A*, it is not difficult to observe that sharp turns still exist in the path (an average of 3.75 times on this occasion). The path generated by the proposed UAVRM-A* algorithm, on the other hand, adjusts the direction of part of the path to accommodate the pathfinding constraints based on traditional A*, successfully avoiding sharp turns while maintaining a small deviation in the overall pathfinding trend. The flight time and outage time are very close to those of traditional A*, with a slightly longer flight time (approximately 4% more) and a significantly shorter outage time (approximately 40% less) compared to D3QN. As for the modeling time, the D3QN algorithm took nearly 45 h to train the model, while the navigation model constructed for A*-based algorithms in this paper could be built in approximately 2 s, sharing the same structure, scale, and granularity as D3QN. It is worth mentioning that the pathfinding of D3QN involves the process of repeatedly loading the trained model, resulting in a significantly longer pathfinding time than the A*-based algorithms. UAVRM-A*, with additional pathfinding constraints compared to traditional A*, also takes slightly longer to perform pathfinding. These results indicate that the proposed UAVRM-A* algorithm significantly reduces the outage duration while maintaining a similar path length compared to D3QN, possesses the capability to integrate UAV flight characteristics, and significantly shortens the modeling time.

**Figure 7:**
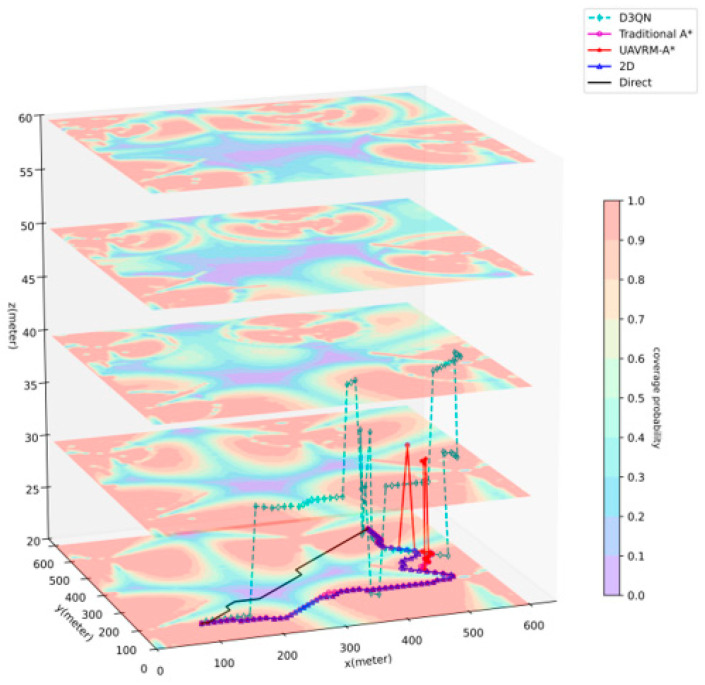
Result of solution for low aerial area.

Figure 7 depicts the path planning results of various methods in the low aerial area, where $z_{\min}=20\;m$, $z_{\max}=60\;m$, $u_{I}=(100,100,20)$, $u_{F}=(500,500,20)$. Table 3 shows the detailed data. In this scenario, a significant number of buildings are present, and they are densely distributed, rendering the overall environment more complex than the previous one. Considering the potential influence of building obstruction to the training, a restriction was imposed to prevent entering buildings, thereby endowing the D3QN with the capability to avoid them. Since a straight line from the start to the goal is obstructed by buildings, the Direct method deviates slightly from this line to bypass the buildings, but similar to the previous occasion, it remains the shortest path with an excessively long outage duration. Given the proximity of the airspace to the base station, radio signals are stronger at a lower height (manifested by higher coverage probability on the radio map), and the 2D method is at the altitude closest to the base station height, leading its theoretical optimal path to be inclined towards Line-of-Sight (LoS) regions. Influenced by this characteristic, traditional A* and UAVRM-A* exhibit slight differences in a few routing choices, but both paths largely coincide, with both slightly outperforming 2D. Traditional A*, lacking the ability to recognize buildings and consider UAV flight characteristics, generates results that include unrealistic phenomena like traversing buildings and excessively large turning angles, compromising its effectiveness. In contrast, UAVRM-A* successfully avoids these issues. When compared to D3QN, D3QN shows the longest flight and outage time, with numerous sharp turns in the path. UAVRM-A* ultimately reduces flight time by approximately 4% and outage time by around 20% compared to D3QN. Considering D3QN’s training characteristics, it can be inferred that in an environment with notably good radio coverage, the advantages of D3QN are diminished, resulting in a relatively modest optimization margin for UAVRM-A*. As in the previous occasion, modeling time consumption remains significantly shorter for A*-based algorithms compared to the model trained by D3QN. These findings not only corroborate the conclusions drawn from the previous occasion but also further validate obstacle avoidance capability for UAVRM-A*. Additionally, it is observed that both D3QN and UAVRM-A* exhibit clear advantages in complex signal distribution environments, whereas in environments with significantly good signals, D3QN gradually falls behind due to its training characteristics, while UAVRM-A* tends to favor LoS regions during pathfinding, leading to a valid but less pronounced optimization effect.

Based on the above comprehensive analysis of the experimental results, the following conclusions can be drawn: In the same scenario, both the D3QN algorithm of DRL and the proposed UAVRM-A* algorithm outperform the 2D method in terms of path planning. The path planning solutions between these two algorithms exhibit high similarity, with UAVRM-A* demonstrating a flight time deviation within 4% and a reduction in outage time by approximately 20% to 40% compared to D3QN. Furthermore, UAVRM-A* aligns with the flight characteristics of UAV, as it avoids sharp turns and building penetrations. It also offers advantages in path planning in environments with complex signal distributions, helping the UAV to approach LoS regions more easily in environments with significantly better radio connection quality. Regarding modeling, under conditions of identical structure, scale, and granularity, the modeling time required for the navigation model corresponding to UAVRM-A* is significantly shorter than that of D3QN training model. This indicates that the proposed UAVRM-A* algorithm can ensure results with minimal deviation from DRL method while optimizing radio communication quality and reducing model construction time, thereby validating the effectiveness of its path planning capabilities.

## 6. Conclusions

This paper delves into the problem of path planning for cellular-connected UAVs based on a 3D radio map. Initially, a navigation model is constructed according to the radio map. Subsequently, an improved A* path planning algorithm, termed UAVRM-A*, is proposed within this model framework. Ultimately, a comparative analysis of the path planning results between the proposed algorithm and the D3QN algorithm from DRL is conducted in a specific dense urban scenario. The results indicate that, under the same scenario and environment, the UAVRM-A* path planning solution aligns with UAV flight characteristics, effectively avoids buildings, and demonstrates superior advantages in environments with complex signal distributions. Compared to D3QN, the UAVRM-A* algorithm yields smaller flight time deviations and significantly reduces radio interruption rates, and the modeling time of the model is notably shorter than the training time required for deep reinforcement learning models. These findings validate the effectiveness of the proposed algorithm’s path planning capabilities, providing a reference for obtaining reasonable and optimal path planning results more swiftly.

To ensure the representativeness of the research, the path planning research primarily focused on scenarios with constant velocity, aiming to simultaneously minimize flight time and radio outage time. However, real-world applications necessitate consideration of more complex scenarios to further enhance algorithm accuracy. Consequently, future work can be summarized as follows:

1. Multi-UAV Path Planning: The algorithm proposed in this paper is designed solely for single-UAV scenarios. In practical dense urban environments, however, multiple UAVs may operate concurrently, potentially requiring cooperative flight. Hence, future algorithms should not only optimize individual paths but also guarantee spatio-temporal conflict-free paths for multiple UAVs. Furthermore, formation flying strategies might be incorporated during cooperative operations to enhance overall system performance.
2. Real-time Replanning in Dynamic Environments: This research primarily investigates static path planning. In reality, dynamic obstacles necessitate UAV capabilities for obstacle detection and dynamic path regeneration. Additionally, while the A* algorithm excels in static path planning within discretized spaces, it faces significant challenges in dynamic path planning scenarios.
3. Comprehensive Optimization Integrating UAV Energy Consumption Models: Actual UAVs incur higher energy consumption during maneuvers such as turns and altitude changes, resulting in non-uniform energy usage even under constant velocity conditions. The proposed algorithm does not explicitly incorporate energy consumption. Integrating UAV energy consumption as a core optimization objective or embedding this constraint within the navigation model still remains a complex challenge for complex network-based approaches.

Therefore, future work necessitates a comprehensive consideration of additional factors, employing more realistic radio maps as the environmental model (validated through field measurements where feasible) and further refining the A* algorithm, enabling it to respond to practical conditions more accurately and quickly.

## Figures and Tables

**Table 1:** Element definitions for sets V(AV,LV) and E(AE,LE).

Element	Description
$v_{i}(a_{i},l_{i})$	The identifier of node $v_{i}$, used to distinguish between nodes.
$a_{i}$	The attribute set of $v_{i}$, storing associated attribute information.
$l_{i}$	The attribute set of node $v_{i}$, storing associated attribute information.
AV	The set of all node identifiers, $a_{i}\in AV$.
LV	The set of all node attribute sets, $l_{i}\in LV$.
$e_{ij}(a_{ij},l_{ij})$	A directed edge $e_{ij}$. Note that two nodes $v_{i},\;v_{j}$ can be connected by two edges $e_{ij},\;e_{ji}$, in opposite directions.
$a_{ij}=(a_{i},a_{j})$	The identifier of edge $e_{ij}$, indicating its direction from $v_{i}$ to $v_{j}$.
$l_{ij}$	The attribute set of edge $e_{ij}$, storing associated attribute information, including but not limited to the weight.
AE	The set of all edge identifiers, $a_{ij}\in AE$.
LE	The set of all edge attribute sets, $l_{ij}\in LE$.

**Table 2:** Detailed result of solution for medium aerial area.

Method	Average Flight Time (s)	Average Outage Time (s)	Average Obstacle Collisions	Average Sharp Turns	Average Modeling Time (s)	Average Pathfinding Time (s)
D3QN	90.60	15.52	0.00	10.00	162,101.77	250.87
Traditional A*	95.25	8.45	0.00	3.75	2.19	0.07
UAVRM-A*	94.71	8.61	0.00	0.00	2.31	0.15
2D	99.35	5.74	0.00	0.00	Not applicable	Not applicable
Direct	56.56	27.18	0.00	0.00	Not applicable	Not applicable

**Table 3:** Detailed result of solution for low aerial area.

Method	Average Flight Time (s)	Average Outage Time (s)	Average Obstacle Collisions	Average Sharp Turns	Average Modeling Time (s)	Average Pathfinding Time (s)
D3QN	92.32	11.01	0.00	8.00	166,739.22	323.86
Traditional A*	87.64	7.97	2.10	0.65	2.19	0.02
UAVRM-A*	88.37	8.03	0.00	0.00	2.26	0.04
2D	88.42	8.32	0.00	0.00	Not applicable	Not applicable
Direct	59.49	24.26	0.00	0.00	Not applicable	Not applicable

## Data Availability

The original contributions presented in the study are included in the article; further inquiries can be directed to the corresponding author.
